# Supplementary material for: Baseline infection prevalence, risk factors and treatment outcomes of visceral leishmaniasis in Northeastern Uganda: A cross-sectional study
Source: PLoS Negl Trop Dis. 2025 Jan 13;19(1):e0012783. doi: 10.1371/journal.pntd.0012783 (PMC11760575; doi:10.1371/journal.pntd.0012783)
Supplement: S1 File — (DOCX) [file pntd.0012783.s002.docx]

**Variable Names and Descriptions:**

1. ID: Unique identifier for each study participant
2. Age_respondent: Age of the respondent (in years)
3. Religion_respondent:

- 0=Catholic
- 1=Moslem
- 2=Anglican
- 3=Others

1. Marital_ status_resp (Marital Status of Respondent)

- 0= Single
- 1= Married
- 2= Divorced
- 3= Widowed

1. Ethnicity_resp (Ethnicity of Respondent)

- 0= Matheniko
- 1= Tepeth
- 2= Turkana
- 3= Bokora
- 4=Others

1. Residence_resp (Residence of Respondent)

- 1= Rural
- 2= Urban

1. Education_resp (Education level of respondent)

- 1= No Education
- 2= Primary
- 3= Secondary
- 4= Postsecondary

1. Income_HH (Monthly Household Income in Uganda Shillings)

- 1= < 50,000
- 2= 50,001 to 100,000
- 3= 100,001 to 200,000
- 4= 200,001 to 500,000
- 5= 500,001 to 3,000,000
- 6= >3,000,000

1. Insectcide_Spray (Do you spray animals in your Kraal with Insecticides?)

- 0= Yes
- 1= No

1. Treated_VL (History of Treatment for VL)

- 0= Yes
- 1= No

1. Family_Treated_VL (Has any Family member been Treated of VL?)

- 0= Yes
- 1= No

1. Travel_ VL-Ende (History of Travel to a VL Endemic Area)

- 0= Yes
- 1= No

1. Termite Mounds (Presence of Termite Mounds around a Homestead)

- 0= Yes
- 1= No

1. Acacia_ Trees (Presence of Acacia Trees in the surrounding)

- 0= Yes
- 1= No

1. Sleeping Space (sleeping space in homestead/surroundings)

- 0= Yes
- 1= No

1. _Bed_nets (Presence of bed nets in homestead/surroundings)

- 0= Yes
- 1= No

1. Rooms_in_HH (Specific number of rooms in the house)
2. Sleeping _Area_type (the place where Participant sleeps: - bed or Ground))

- 0=Bed
- 1=Ground
- 2=Changes depending on the season

1. MUAC_Score (Numerical value of each participant’s Mid Upper Arm Circumference)
2. Malaria (Malaria Test Status of Participant)

- 0=Negative
- 1=Positive

1. Health_Worker_VL (Do you have any health worker you know, that offers you information about VL in this community)

- 0= Yes
- 1= No

1. Outreach_VL (Have you attended any outreach in your community where VL was discussed?)

- 0= Yes
- 1= No

1. Information_Signs_VL (Do you have a friend in your community who has talked to you about the signs, symptoms, and prevention of VL?)

- 0= Yes
- 1= No

1. Belief_Hospital_VL (Do you believe the hospital has adequate capacity to treat and manage VL if you acquire an infection?)

- 0= Yes
- 1= No

1. VL_chances; (Compared to your age mates, what are your chances of getting VL?

- 1=Low
- 2=Average
- 3=High

1. VL_Worst (Compared to other diseases, do you think VL is the worst disease one can get?)

- 1=Yes
- 2=No

1. Fever (Do you have Fever?)

- 0= Yes
- 1= No

1. Length_Fever (For how long have you had a fever?)

- 0=< 2weeks
- 1=2-4 weeks
- 2=>4 weeks

1. Abdomen_ swell (Does your Abdomen Swell?)

- 0= Yes
- 1= No

1. Weight_ Loss (Have you lost weight?)

- 0= Yes
- 1= No

1. Family_ size (A specified number was entered)
2. Weight (Weight in kilograms entered)
3. Age_Participant (in years)

- 1=≤5 years
- 2=6-11 years
- 3=12-17 years
- 4=18-23 years
- 5=24-29 years
- 6=30-35 years
- 7≥36 years

1. Nutrition_Status

- 1=Normal
- 2= Moderately Malnourished
- 3= Severely Malnourished

1. rk39_results

- 0=negative
- 1=Positive

1. County1

- 1=Matheniko
- 2=Tepeth
- 3=Moroto Municipality

1. Subcounty1

- 1=Katikekile
- 2=Lotisan
- 3=Nadunget
- 4=Rupa
- 5=Tapac
- 6=Loputuk

1. Parish1

- 1=Acherer
- 2=Katikekile
- 3=Mogoth
- 4=Musupo
- 5=Nakiloro
- 6=Narenkegu
- 7=Naturumrum
- 8=Rupa
- 9=Tapach

1. Profession_resp1(Occupation of respondent)

- 1=Farming
- 2=Professional (formal employment)
- 3=Manual Labour
- 4=Sales and Services
- 5= Cattle rearing
- 6=Others
- 7=Cattle rearing & Farming

1. newVL_transmission (Knowledge of VL transmission)

1= knows

2=Do not know

1. newsex:(Gender of the participant)
   - 1 = Female
   - 2 = Male
2. newOwns_mosquito_net: (Whether the participant owns and uses a mosquito net)
   - 1 = Yes
   - 2 = No
