# Supplementary material for: Baseline infection prevalence, risk factors and treatment outcomes of visceral leishmaniasis in Northeastern Uganda: A cross-sectional study
Source: PLoS Negl Trop Dis. 2025 Jan 13;19(1):e0012783. doi: 10.1371/journal.pntd.0012783 (PMC11760575; doi:10.1371/journal.pntd.0012783)
Supplement: Attachment — Submitted filename: DETAILED LIST OF YOUR RESPONSES TO THE REVIEW COMMENTS.docx [file pntd.0012783.s003.docx]

[drsagaki@yahoo.com](mailto:drsagaki@yahoo.com)

Dated: 18/09/2024

Dear Reviewers,

RE: **DETAILED LIST OF YOUR RESPONSES TO THE REVIEW COMMENTS**

Greetings!

I thank you so much for sparing your valuable time to review this article. I would wish to let to your attention that these comments have been very impactful to this manuscript and have been taken positively.

Kindly receive them, in this tabulated form below.

| **Statement in the original manuscript** | **Comment** | **Response** | **Location of response in the revised manuscript** |
| --- | --- | --- | --- |
|  | Reviewer #1: The cross-sectional study aims to describe the prevalence of VL pre-elimination, associated factors, and treatment outcomes in Moroto District, Uganda. Some major issues need to be considered for revision. | This is to thank you for your time, spared for reviewing and commenting on our work.  The issues identified have been revised. | Entire document |
| In Abstract:  Under Result section (Page 2, line number 36)…. The average hospitalization for VL was 17 days…  In Abstract:  Under Conclusion section (Page 2, line number 40) …. Patients with VL were hospitalized for not more than 17 days. | - Abstract: In the results section, authors declare that "The average hospitalization for VL was 17 days", implying that some patients have longer lengths of stay. However, in the conclusion, they say, "Patients with VL were hospitalized for not more than 17 days". Please clarify such inconsistency | Thank you so much for this observation. It was indeed erroneously stated. It has been corrected to read as “The average hospitalization for VL was 17 days” in both the results and conclusion sections of the abstract. | Page 2, line number 40 |
| Under Introduction section (Page 2, line number 47) ... in areas where studies have been conducted…) | - Introduction, page 3, line 4: Remove the phrase "in areas where studies have been conducted." | The phrase "in areas where studies have been conducted." has been removed | Page 2 |
| Under Introduction section (Page 2, line number 48 to 51) …in Low- and Middle-Income Countries (LMIC) like Bangladesh, Ethiopia, India, Nepal, South Sudan, Sudan, and Brazil. These countries account for more than 95% of the reported burden (Jervis *et al.* 2017), but may be an underestimate since data on the disease are largely considered incomplete… | - Introduction, page 3, lines 5/6: This set of countries no longer accounts for 95% of cases worldwide; the reference is outdated. Kenya and Somalia are among the most affected countries today, while Bangladesh and Nepal are out. | Indeed, you are very correct. We appreciate this input. We searched and found up-to-date literature describing the global distribution of VL. We have therefore corrected the statement to read as “Visceral leishmaniasis poses a serious global health challenge, with approximately 50,000 to 100,000 new cases reported each year, primarily in endemic regions across South Asia, East Africa, and parts of South America, in countries like Brazil, **Ethiopia, Kenya, Somalia, South Sudan, Sudan** and India (World Health Organization, 2023)” | Page 2, line 48 to 51 |
| Under Introduction section (Page 2, line number 51 to 52) …. Evidence shows that the disease peaks during the sunny and warm pleasant seasons (Jervis *et al.* 2017)… | - Introduction, page 3, lines 10/11: certainly, more than than ref; references too old. | The old references have been deleted. Recent Literature containing similar data has been added. | Page 2 to 3, line numbers 48 to 62 |
| In the Title section (Page 1 Line number 1) and under Abstract section (Page 1 line number 21 to 22)  "pre-elimination prevalence…” | The authors use the term "pre-elimination prevalence" in the title, abstract, and throughout the text, suggesting that some effort is being made to eliminate VL from this region. However, there is no evidence in the text that a formal elimination program is ongoing. Therefore, it would be better to refer only to "prevalence" or "baseline prevalence." | Yes, some efforts like community mobilization and sensitization on case definition for detection and early referral of cases, active VL case search, diagnosis, and treatment are currently ongoing in Moroto district. | Page 13, line number 288 to 291 |
| Under sample size section, Page 5 lines 123 to 141 | The parameters used for sample size selection need to be given. The author presents a formula for the number of clusters, fixing the number of 24 respondents in each cluster. We need to know the estimated prevalence of VL in the Moroto district (p) to evaluate whether the final sample size (n=288) is adequate | The parameters used for sample size determination have been given as seen below: -  The participants were sampled in a multistage cluster sampling. The final cluster was the manyatta and by using a formula of cluster surveys, a total of 12 clusters (manyatta) were used for this study as used elsewhere.  The number of clusters was derived from the formula below: -  C= p (1-p) D  S^2^b  Where, C: is the number of clusters selected (24)  p: is estimated prevalence of VL in Moroto district (17.2% as per Odoch’s study in Amudat district)   D: Design effect and 1.3 was used in this study because it has been used in similar study design of VL in a community survey in Uganda. b: Number of respondents per cluster (manyatta) (b=24)   S= standard error  S=(CI/Cα)  CI=width of desired confidence interval (CI=0.05)  Cα =value of standard normal curve corresponding to accepted α -value of 1.96  C= 0.172(1-0.172) 1.3  24(0.05/1.96)^2^  Sample size = Number of clusters (12) X number of participants per cluster (24).  This gives 288 participants. | Page 5-6, line number 128 to 149 |
| Under sample size section, (Page 5 lines 123 to 141) | - Regarding sample size, there is also a need for a better justification for using a design effect of 1.3. Authors say this value was "used in similar study design of VL in a community survey in Uganda (Odoch & Olobo 2013)." Consulting such a study, we can see that they used the value of D=1.3 based on a study in a cluster randomized trial in Brazil. There is no reason to believe that the value calculated in a different context would apply in the Moroto district. With the actual results of this study, the author may check if such an assumption is correct. | We appreciate the observation. However, we searched but we failed to get a study within the same context as in Moroto district, other than the one by Odoch, which was conducted from Amudat district, which neighbors Moroto district. Additionally, a design effect of 1.3 has a high-power level of about 0.7 (Hernán et al., 2019) | Page 5 line numbers 136 to 139 |
| Under the Data collection techniques (Page 7, line numbers 169 to 171)…Data collection is a process of gathering and measuring information on targeted variables in a systematic way (Creswell, 2003). This process requires appropriate techniques tailored to the kind of data required and categories of people providing the data…. | - Data collection techniques: Please remove the first paragraph. This is unnecessary in this context. | Thank you so much for this observation. We have reviewed and found unnecessary. We have therefore deleted it. | Page 7, line numbers 179 to 181 |
| Under Data analysis section (Page 8, line numbers 208 to 210) | - Data analysis: Please make sure to indicate if sampling weights were used to correct estimates for complex sampling procedures. Explain how "clustering was factored in at county/constituency level" was done. Did you use multilevel or GEE approaches? | We appreciate your observation. However, we clearly understand that the primary purpose of using sampling weights is to correct for any biases that may arise from non-random sampling methods. Random sampling was conducted at Parish, Village, Manyatta, and at household level.  To factor in clustering at the county level during binary logistic and multivariate logistic regression analysis, the following steps were used; data structure was understood, a suitable model was selected, and we incorporated clustering. A mixed-effects **model was used** to cater for random effects for counties, which account for variation at the county level. We interpreted the results and reported them. | Page 6, line numbers 153 to 165  Page 8, Line numbers 220 to 224 |
| Under Data analysis section (Page 8, line number 198) | - Data analysis: please make clear what does "(plus the ones I suggested)" mean. | Thanks for the observation. "(plus the ones I suggested)" has been deleted. | Page 8, line number 208 |
| Page 8, line number 211 to 212)…Ethical Approval was obtained from the Research and Ethics Committee (REC) in Mbale Regional Referral Hospital… | - Ethics: Please indicate the number of ethical approval documents and submit a copy. | The REC approval number is: MRRHREC-OUT-011/2020  The REC approval letter has been attached accordingly. | Page 9, line number 226 |
| Result section | - Please indicate how nutritional status and knowledge of transmission was assessed. | Nutritional status was assessed using Mid Arm Circumference measurements (MUAC). Knowledge about VL transmission was assessed by asking Participants about their knowledge on the way VL is transmitted. If they chose the option of a bite by a sandfly then we assumed, they were knowledgeable about transmission. Those who ticked otherwise were not knowledgeable. | Page 11, line numbers 255 to 259 |
| Under discussion section, Page 13, line numbers 297 to 301 | - Discussion, 3rd paragraph: The fact that you had more females in the sample cannot explain the higher sex ratio of leishmania infection among women because prevalences are calculated conditional on sex. | Thank you for this observation. It has made us view the results at a different angle. The higher sex ratio of visceral leishmaniasis (VL) infection among females, with a female-to-male ratio of 3:2, present a notable shift from earlier research that reported a male-dominant ratio of 1:3 (Mwebesa, 2019). This change may indicate evolving epidemiological patterns influenced by various factors, including environmental, social, and behavioral dynamics.  For instance, the increased prevalence among females could be attributed to heightened exposure due to differing roles in household and agricultural activities, particularly in endemic regions. Additionally, it raises important questions about the biological and immunological factors that may predispose women to a higher infection rate in this context.  Further research is needed to explore these dynamics in depth and assess how such changes might affect public health strategies aimed at managing and preventing VL. Understanding the reasons behind this shift could lead to more effective interventions tailored to the specific needs of different populations. Overall, this emerging trend underscores the importance of continuously monitoring gender-specific health outcomes in infectious diseases. | Page 14 to 15, lines 317 to 330 |
| Under the discussion section, Page 14, line numbers 311 to 317 | - Discussion, malaria: explanation is not adequate. You found a strong association between positivity to malaria and the prevalence of infection, although not significant (probably due to the insufficient statistical power of the study). | We thank you for your input. We however designed our study to have enough statistical power, based on the design effect we used. We do not think that the plausible explanation for the observed findings was due to the less statistical power of the study. We have reviewed the literature and discussed these findings from a different angle as seen below; Given that malaria is known to impair the immune response, one might expect that individuals with malaria could be more susceptible to VL due to the compromised immune system (van den Bogaart et al., 2012).  The lack of statistical significance in this association does not diminish its public health relevance. It may reflect underlying complexities in the interactions between these diseases. For example, individual variability in immune responses, age, sex, or co-existing health conditions could influence susceptibility and disease progression (Kouadio et al., 2020). Additionally, the timing and severity of malaria infections could affect how the immune system responds to leishmaniasis, potentially obscuring clearer associations.  Environmental factors prevalent in Moroto, such as vector habitats and socio-economic conditions, may also contribute to the observed relationship. Areas with high malaria transmission often share ecological features with those conducive to VL transmission, which could result in overlapping risk factors (Murray et al., 2017). Furthermore, behavioral aspects, such as outdoor activities that increase exposure to both vectors, may enhance the likelihood of co-infection. | Page 15-16, line numbers 340-359 |
| Under discussion section, Page 14, line numbers 318 to 322 | - Discussion, malnutrition: Please explain and provide a reference for the sentence "malnutrition decreases the likelihood of diagnosis of asymptomatic VL infection." | Thank you for this observation. An in-depth explanation of the phrase has been given: - The statement "malnutrition decreases the likelihood of diagnosis of asymptomatic VL infection" indicates that malnutrition can impact the clinical presentation and detection of visceral leishmaniasis (VL) in individuals who do not show overt symptoms. Malnourished individuals often experience impaired immune function, which can lead to atypical or milder presentations of infections, making asymptomatic cases less likely to be identified. Furthermore, malnutrition may affect health-seeking behavior, as individuals with limited resources might not pursue medical attention unless symptoms are severe.  Research has shown that malnutrition compromises the immune system, reducing the body's ability to mount effective responses to infections, which may alter the detection rates of asymptomatic cases of VL (Alvar et al., 2012). This underscores the need for heightened awareness and targeted diagnostic approaches in malnourished populations to improve identification and management of asymptomatic VL. | Page 16, line numbers 362 to 372 |
|  |  |  |  |
|  | Reviewer #2: (No Response) |  |  |
|  |  |  |  |
|  | Reviewer #3: In this manuscript the the authors evaluated the Prevalence, Risk Factors and Treatment Outcomes of Visceral Leishmaniasis in Northeastern Uganda. TThe situation discussed is of utmost importance to better understand how VL behaves in different regions. However, the manuscript needs to be greatly improved to make it more robust and with more precise information about the topic addressed. Here are my considerations. | Thank you for sparing your time to review this manuscript. |  |
| Under discussion section, Page 1, line numbers 1 to 2… Pre-elimination Prevalence, Risk Factors and Treatment Outcomes of Visceral Leishmaniasis in Northeastern Uganda: A cross-sectional study | 1) Title. I suggest you review the title, as I believe that the current format (Pre-elimination...) does not make it clear that the disease was subsequently eliminated or if any action was implemented to that end. | Some efforts like community mobilization and sensitization on case definition for detection and early referral of cases, active VL case search, diagnosis, and treatment are currently ongoing in Moroto district. | Page 13, line number 288 to 291 |
| Under Abstract section, Page 2, line numbers 34 to 36… | 2) Abstract The authors mention that patients with VL presented the following symptoms: fever, headache, abdominal pain and swelling, coughing, night sweats, diarrhea, fatigue, breathlessness, and nose bleeding. However, this information is not included in the results of the manuscript. | Thank you for this observation. VL symptoms among the VL patients had been erroneously left out. However, this has been corrected: - “The 11 patients who reached the treatment Centre, presented with following symptoms: - fever (81.82%), headache (72.72%), abdominal swelling (36.36%), abdominal pain (72.72%), diarrhea (36.36%), night sweats (63.63%), fatigue (63.63%), cough (63.63%), breathlessness (45.45%), and nose bleeding (9.09%).” | Page 13, line numbers 275 to 278 |
| Under Data collection techniques (Page 7  Under Data analysis section (Page 7) | 3) Methodology:  a) It is unclear how malnutrition was measured. b) It is unclear how symptoms and signs of the disease were assessed. | Nutritional status was assessed using Mid Arm Circumference measurements (MUAC).  The signs and symptoms of VL for patients who reached the treatment Centre were assessed by ticking against a list of common VL symptoms and signs, which are in a form available at the treatment Centre. | Page 11, line numbers 255 to 259  Page 7, line numbers 192 to 194 |
| Under Results section (Page 7, line number 221) | 4) Results. The results presented are a bit confusing. Some numbers do not agree. I suggest reviewing point by point as follows: a) Some data are not in agreement. The authors report that there were 34 single people, however only in the age group of 1 to 4-5 years were 59 participants included and in the age group of 6 to 11 years 37 participants were included. I suggest reviewing the data or the way of writing these results. | Thank you so much for your input. This is clarification on this input: - In this research, not all respondents were participants and not all participants were respondents. Respondents were those who answered the questionnaire. In this case, marital status was for the respondents. This makes the figure presented to tally. Kindly when you re-examine those results, you find that the results were for those of respondents and participants. | Page 9, line number 237 |
| Under Results section (Page 7, line number 222) | b) Regarding the prevalence of infection, which age group was most affected? This is not clear. Furthermore, participants with positive results presented some symptoms of the disease. This is described in the abstract, but this information is not included in the results. Regarding the prevalence of infection, I ask whether patients were tested for HIV infection. This information is important, since it is known that serological tests have lower sensitivity in this population. | Thank you for this observation.  The distribution of VL infection across age group is as follows; Of the 15 individuals found to have VL, 6 (40.0%) were aged 3Months -17 years, 3 (20.0%) were aged 18-29 years, and 6 (40%) were aged 30 years and above.  Thank you for this observation. VL symptoms among the VL patients had been erroneously left out. However, this has been corrected: - “The 11 patients who reached the treatment Centre, presented with following symptoms: - fever (81.82%), headache (72.72%), abdominal swelling (36.36%), abdominal pain (72.72%), diarrhea (36.36%), night sweats (63.63%), fatigue (63.63%), cough (63.63%), breathlessness (45.45%), and nose bleeding (9.09%).”  All VL patients are tested for HIV before initiation of treatment, because the first line treatment for VL (Sodium Stibogluconate combination with Paromomycin) is contra-indicated among HIV/VL co-infected patients. | Page 11, line numbers 245 to 248  Page 13, line numbers 275 to 278 |

Looking forward to hearing from you,

Yours sincerely

Patrick Sagaki
