# Supplementary material for: Baseline infection prevalence, risk factors and treatment outcomes of visceral leishmaniasis in Northeastern Uganda: A cross-sectional study
Source: PLoS Negl Trop Dis. 2025 Jan 13;19(1):e0012783. doi: 10.1371/journal.pntd.0012783 (PMC11760575; doi:10.1371/journal.pntd.0012783)
Supplement: Attachment — Submitted filename: Response to Reviewers.docx [file pntd.0012783.s004.docx]

[drsagaki@yahoo.com](mailto:drsagaki@yahoo.com)

Dated: 8/12/2024

Dear Editors & Reviewers,

RE: Responses to Editors’ & Reviewers’ comments

Greetings!

Thank you so much for sparing your valuable time to review this article. I wish to inform you that these comments have been very impactful to this manuscript and have been taken positively.

Kindly receive our responses, in the table below:-

| **Statement in the original manuscript** | **Comment** | **Response** | **Location of response in the revised manuscript** |
| --- | --- | --- | --- |
| Pre-elimination | Please change the title to 'Baseline infection prevalence' instead of Pre-elimination unless there are concrete and official plans at the government level to eliminate VL from Northeastern Uganda | The title “Pre-elimination Prevalence, Risk Factors and Treatment Outcomes of Visceral Leishmaniasis in Northeastern Uganda: A cross-sectional study” has been changed to “Baseline Infection Prevalence, Risk Factors and Treatment Outcomes of Visceral Leishmaniasis in Northeastern Uganda: A cross-sectional study  ” | Line number 1 page 1 |
| No study limitation | Add a paragraph on the limitation of the study in the discussion | The Study limitation has been included.  The study faced two main limitations: incomplete follow-up and treatment availability issues. A significant portion of VL-infected individuals (26.67%) did not reach the treatment center, which may introduce bias in assessing treatment outcomes, as non-responders' outcomes could differ from those who sought treatment. Additionally, stockouts of AmBisome (a second-line treatment) at the treatment center limited its availability to some patients, highlighting gaps in healthcare infrastructure that could affect the generalizability of the treatment results to other settings. | Line 399 to 406, Page 17 |
| After meeting the selected participants to take part in the study, the research team introduced themselves, explained the purpose of the study and sought for written consent from the participants. For child participants, written formal consent was obtained from their parents/guardians. After receiving their consent, the research team administered the questionnaires to the participants. | 1) Thank you for including an Ethics Statement for your study. Please include:  i) A statement that formal consent was obtained from all participants (must state whether verbal/written) OR the reason consent was not obtained (e.g. anonymity). | After meeting the selected participants to take part in the study, the research team introduced themselves, explained the purpose of the study and sought for written consent from **all** participants. For child participants, written formal consent was obtained from their parents/guardians. After receiving their consent, the research team administered the questionnaires to the participants. | Line 179 to 182, Page 7 |
| No list of legends for Stata data set | 2) We have noticed that you have uploaded Supporting Information files, but you have not included a list of legends. Please add a full list of legends for your Supporting Information files after the references list. | List of legends for the stata data set has been added. | Line 523 to 688 page 22 to page 25 |

Looking forward to hearing from you,

Yours sincerely

Patrick Sagaki
